# Benign Paroxysmal Positional Vertigo (BPPV) in COVID-19

**DOI:** 10.3390/audiolres11030039

**Published:** 2021-08-13

**Authors:** Pasqualina Maria Picciotti, Giulio Cesare Passali, Bruno Sergi, Eugenio De Corso

**Affiliations:** 1 Otolaryngology Institute-Department of Head and Neck, Fondazione Policlinico Universitario A Gemelli IRCCS, 00168 Rome, Italy; GiulioCesare.Passali@unicatt.it (G.C.P.); bruno.sergi@unicatt.it (B.S.); eugenio.decorso@policlinicogemelli.it (E.D.C.); 2 Otolaryngology Institute, Università Cattolica del Sacro Cuore, 00168 Rome, Italy

**Keywords:** COVID-19, vertigo, vestibular, BPPV

## Abstract

Objective: The purpose of this article is to describe BPPV in COVID-19 patients by discussing the possible mechanisms underlying the onset of this vertigo. Methods: We studied eight patients (4 F, 4 M, aged between 44 and 69 years) with COVID-19 infections complaining of vertigo. Patients were evaluated at the end of infection with an accurate clinical history, and the investigation of spontaneous, positional and positioning nystagmus. Results: The vestibular findings showed benign paroxysmal positional vertigo (BPPV) in all the patients. Three patients had a mild phenotype of the COVID infection, whereas five subjects were hospitalized for the COVID infection and in three cases intensive care was required. Vestibular evaluation showed an involvement of posterior semicircular canals in five patients and horizontal in three. Three patients were treated with the Epley maneuver, two with Semont, one with Lempert and two with Gufoni maneuvers. Conclusions: We hypothesize that BPPV in COVID-19 infections can be relate to drugs, prolonged bed rest and to direct damage by viral infection on the peripheral vestibular system and in particular on the otolitic membrane due to the cytopathic effect of the virus and to the inflammatory response. Studies on large series of patients are needed to confirm our preliminary observation and to better evaluate the pathophysiological mechanisms underlying BPPV in these patients.

## 1. Introduction

In December 2019, a novel type of β-coronavirus, SARS-CoV-2, named COVID-19 by the World Health Organization (WHO), was identified as the cause of a cluster of pneumonias in the city of Wuhan, Hubei Province of China. Since then, the understanding of COVID-19 pathophysiology and clinical presentation has been ever evolving.

Pneumonia is the most frequent and the most serious manifestation, presenting as fever, cough, dyspnea and bilateral infiltrates on chest imaging. However, there have been many other presentations, including conjunctivitis, upper respiratory symptoms, cardiomyopathy, deep venous thrombosis and even ischemic stroke. At least one neurological symptom has been reported in over 90% of patients, and patients with severe COVID-19 infection develop more neurological abnormalities in comparison to those with mild infection. Headache, confusion and dizziness are the most commonly referred symptoms [1].

Benign paroxysmal positional vertigo (BPPV) is the most common cause of peripheral vestibular dysfunction, with an incidence of 0.6% per year and a highly variable prevalence of 10.7–64/100.000 [2]. The pathophysiological mechanism can be described by two theories: “cupulolithiasis” and “canalolithiasis”. BPPV is idiopathic (I-BPPV) in 35% of cases, while secondary BPPV can be related to traumatic brain injury, Ménière’s disease, vestibular neuritis, otologic and non-otologic surgery, herpes zoster oticus and inner ear ischemia. Clinically, patients report an abnormal sensation of motion and/or rotational vertigo, usually lasting less than one minute, generally induced by a change in the position of the head [3]. BPPV mostly develops in the posterior (PC) and horizontal (HC) semicircular canals. PC-BPPV accounts for 60–90% of all BPPV cases, while HC-BPPV accounts for 5–30% [2]. BPPV is idiopathic (I-BPPV) in 35% of cases [4]. Secondary BPPV can be related to traumatic brain injury (TBI), Ménière’s disease, vestibular neuritis, otologic and non-otologic surgery, prolonged bed rest, herpes zoster oticus and inner ear ischemia.

The aim of this paper is to describe BPPV in COVID-19 patients by discussing the possible mechanisms underlying the onset of vertigo.

## 2. Methods

This is a retrospective evaluation of eight medical records, collected over a 4-month period, between June and October 2020, referring to patients with COVID-19 infections and vertigo without audiological symptoms. At the time of the study in our post-COVID-19 ENT service, we evaluated an average of 5.5 patients per week, which in the period considered consists of about 110 patients. Eight subjects considered in this study (7.2% of the total) were aged from 44 to 69 years (4 females and 4 males), and they were referred to the vestibular service of our institution, in the day clinic.

Patients were evaluated at the end of infection after the negative result of a reverse transcription–polymerase chain reaction (RT-PCR) test.

All patients were evaluated with an accurate clinical history, including standard questions about their COVID-19 infection, associated diseases and pharmacological therapies. We also asked the patients about the start of the vertigo, during or after COVID-19 infection.

Spontaneous, positional and positioning nystagmus using Frenzel glasses and video-oculography were investigated.

The diagnosis of BPPV was based on:

- the history of brief episodes of vertigo induced by rolling the head from side to side while supine;
- the presence of a linear horizontal nystagmus toward the lower ear when the head of the supine patient was rapidly turned from side to side, beating stronger toward the affected ear in geotropic type and on reverse in the apogeotropic ones, with short latency, poorly fatigable and lasting 30 to 90 s (horizontal semicircular canal involvement);
- the presence of a brief latency, torsional, geotropic and paroxysmal nystagmus, with a duration that was usually not overcome in one minute; and direction reverse when the patient comes up to the sitting position (posterior semicircular canal involvement).

## 3. Results

During the COVID-19 pandemic, we observed eight patients affected by COVID infection and vertigo. The vestibular findings were indicative of benign paroxysmal positional vertigo (BPPV) in all the patients. Three patients had a mild phenotype of COVID infection, whereas five subjects were hospitalized for the COVID infection and in three cases intensive care was required. No patients were referred for a previous history of vertigo or deafness.

All patients experienced rotatory vertigo any time they tilted or changed the position of the head, associated with nausea, vomiting and unsteadiness.

Vestibular evaluation showed a torsion upbeat nystagmus in the Dix-Hallpike (DH) and/or Semont maneuvers when cupulolithiasis involved the posterior semicircular canal (PSC) towards the left or right. On the other hand, in canalolithiasis of the horizontal semicircular canal (HSC), we observed a horizontal nystagmus toward the lower (geotropic) or upper ear (apogeotropic) ear when the head of the supine patient was rapidly turned from side to side.

Patients were treated with specific repositioning maneuvers and reassessed after seven days, until the resolution of symptoms and nystagmus was achieved. The Epley maneuver was performed in three patients, Semont in two and Lempert and Gufoni only in one patient as reported in Table 1. 

## 4. Discussion

Since the emergence of COVID-19, due to its rapid spread and being a serious threat to human health, researchers have made great efforts to understand its pathogenetic characteristics and clinical presentation, and this knowledge is actually ever-evolving.

Although respiratory pathologies have been the major complications of a COVID-19 infection, other presentations such as abdominal pain, deep venous thrombosis, cardiomyopathy and even acute cerebrovascular ischemic attacks have been reported.

Few studies are reported in the literature about vestibular dysfunction associated with COVID-19. Papers often describe case reports or only investigate symptoms.

In this regard, a multicentric Italian study was conducted on 185 COVID-19 patients using an online questionnaire to identify the presence of tinnitus and balance disorders. The authors showed that 18.4% of subjects interviewed reported equilibrium disorders, 94.1% reported dizziness and 5.9% acute vertigo attacks [5]. On the other hand, Korkmaz et al. showed that the rate of vestibular symptoms was 31.8% for dizziness and 6% for true vertigo (6%) [6]. In two large case series from Wuhan, Chen et al. described dizziness in 8% of 274 confirmed COVID-19 patients [7], while Mao et al. reported dizziness in 16.8% of 214 patients [8]. A study from a network of Chicago area hospitals on 509 patients found dizziness in 29.7% of cases at any time during COVID-19 infection [9].

Finally, from the reviews on the topic of dizziness it emerges that there is an important variability among studies [10], and even if dizziness is not uncommon in COVID-19, the studies are not sufficiently specific in order to define its vestibular origin [11].

About clinical reports, two cases of vestibular neuritis have been reported by Malayala and Raza [12] and Vanaparthy et al. [13]. Another two cases have been described by Mat et al. [14] with an instrumental vestibular diagnosis. With a more specific and instrumental study on 41 patients, Gallus et al. described vestibular symptoms, demonstrating that 8.3% of patients experienced dizziness, only one subject experiencing spinning vertigo and dynamic imbalance, and 6.3% experienced static imbalance. They also showed that symptoms regressed and vHIT gain were within the normality range in all post-COVID-19 patients [15].

Furthermore, despite the cited studies, COVID-19-related BPPV has not been described in the literature, even if it is the most common cause of peripheral vestibular vertigo. We believe there may be a close relationship between COVID-19 and vertigo.

What is the exact cause of this relationship? We hypothesize that drugs and inflammation could induce decalcification, damaging the otoconia, and causing BPPV in COVID-19, as described in our previous paper [3]. Alternatively, in cases of more serious infection, with hospitalization and intensive care recovery, immobilization and prolonged bed rest could lead to the development of BPPV. Another hypothesis could be related to the endothelial dysfunction involving cerebral venous hemodynamics; this mechanism is extremely interesting and it was also described for sudden hearing loss [16]. Finally, similarly to what happens in the central nervous system often involved in COVID-19 [17], a direct effect of viral infection on the peripheral vestibular system, namely the otolitic membrane, could justify the onset of vertigo. In this topic, a direct cytopathic effect of the virus, an inflammatory response, a cytokine storm or a vascular event could be proposed as pathogenetic factors.

A final consideration concerns the unusually high proportion of horizontal canal involvement in our series compared to the typical distribution of BPPV subtypes. We found horizontal canal involvement in two out of three patients admitted to intensive care. As it is well known, these patients are often placed in pronation to improve oxygenation, and this forced position could modify the course of positional vertigo, justifying a greater incidence of forms involving the horizontal canal in our series.

In conclusion, further studies are necessary to investigate the effects of COVID-19, as well as for understanding long-term risks, on the vestibular system. In particular, studies on a large series of patients are needed to better evaluate the prevalence and pathophysiological mechanisms underlying BPPV in these patients. The topics of future investigation can make use of instrumental techniques that allow for better study of the involvement of different components of vestibular system.

## Figures and Tables

**Table 1:** (Patients’ data) (Legend: Hosp = hospitalization; IC = intensive care; OV: onset vertigo; maneuver; type and number of sessions).

Cases	Age	Sex	Hosp	IC	Canal	OV	Maneuver
1	48	F	No	No	PSC right	after	Epley (3)
2	44	F	Yes	No	PSC right	during	Epley (1)
3	52	F	No	No	PSC left	after	Epley (1)
4	67	F	Yes	No	HSC (apogeo) right	during	Lempert (3)
5	65	M	Yes	Yes	PSC right	after	Semont (2)
6	68	M	No	No	PSC left	during	Semont (1)
7	59	M	Yes	Yes	HSC (geo) left	after	Gufoni (1)
8	69	M	Yes	Yes	HSC (geo) right	after	Gufoni

## Data Availability

Not applicable.
